# Primary Cilia Restrain PI3K-AKT Signaling to Orchestrate Human Decidualization

**DOI:** 10.3390/ijms232415573

**Published:** 2022-12-08

**Authors:** Bo Li, Ya-Ping Yan, Chen Liang, Yu-Ying He, Ying Wang, Meng-Yuan Li, Si-Ting Chen, Yue Li, Ai-Xia Liu, Gui-Jun Yan, Zeng-Ming Yang

**Affiliations:** 1 College of Veterinary Medicine, South China Agricultural University, Guangzhou 510642, China; 2 College of Animal Science, Guizhou University, Guiyang 550025, China; 3 Department of Reproductive Endocrinology, Women’s Hospital, Zhejiang University School of Medicine, 1 Xueshi Road, Hangzhou 310006, China; 4 Center for Reproductive Medicine and Obstetrics and Gynecology, Nanjing Drum Tower Hospital, Nanjing University Medical School, Nanjing 210008, China

**Keywords:** primary cilium, decidualization, Aurora A, AKT

## Abstract

Endometrial decidualization plays a pivotal role during early pregnancy. Compromised decidualization has been tightly associated with recurrent implantation failure (RIF). Primary cilium is an antenna-like sensory organelle and acts as a signaling nexus to mediate Hh, Wnt, TGFβ, BMP, FGF, and Notch signaling. However, whether primary cilium is involved in human decidualization is still unknown. In this study, we found that primary cilia are present in human endometrial stromal cells. The ciliogenesis and cilia length are increased by progesterone during in vitro and in vivo decidualization. Primary cilia are abnormal in the endometrium of RIF patients. Based on data from both assembly and disassembly of primary cilia, it has been determined that primary cilium is essential to human decidualization. Trichoplein (TCHP)-Aurora A signaling mediates cilia disassembly during human in vitro decidualization. Mechanistically, primary cilium modulates human decidualization through PTEN-PI3K-AKT-FOXO1 signaling. Our study highlights primary cilium as a novel decidualization-related signaling pathway.

## 1. Introduction

Endometrial decidualization is fundamental for successful establishment of human pregnancy [1]. During decidualization, there is a transformation process involving the transformation of mesenchymal endometrial stromal cells into epithelial-like secretory decidual cells [2]. In humans, continuous progesterone surge and intracellular cAMP are essential to induce the decidualization process [3]. Decidualization is modulated by multiple signaling pathways, such as Hedgehog, WNT, and TGFβ/BMP pathways [4,5,6,7]. Impaired endometrial decidualization is often associated with impaired implantation, preeclampsia, recurrent pregnancy loss, and unexplained infertility [3,8,9]. Recurrent implantation failure (RIF) is defined as clinical pregnancy failure after going through more than three embryo transfer cycles with the transfer of a total of at least four good-quality embryos [10]. There is considerable evidence indicating that recurrent implantation failure (RIF) is principally due to impaired decidualization [11]. Decidualization failure remains one of the principal and challenging causes of infertility [12].

Primary cilia are immotile cilia with 9 + 0 configuration that can serve as an ‘antenna’ to transduce many external environment signals, such as canonical Hedgehog, WNT, and TGFβ/BMP pathways [13]. Although primary cilia are non-motile, they are dynamic structures. The assembly and disassembly of primary cilium are balanced by intraflagellar transport machinery tightly linked with cell cycle progression [14]. During the G1 phase, the mother centriole docks at the cell cortex and nucleates a cilium [15]. During the course of the S phase, primary cilia are resorbed following cell cycle reentry to allow centrosomes to engage in mitosis [16]. Ciliary disassembly in vertebrate cells is promoted through HDAC6 phosphorylation by HEF1 and Aurora A at the basal body of primary cilia [17]. Trichoplein (TCHP), a keratin filament-binding protein, can bind and activate Aurora A at the centrioles to block ciliary assembly [18]. Malfunction of primary cilia leads to ciliopathies, such as Joubert syndrome, Polycystic kidney disease, and Meckel syndrome [19]. Based on single-cell transcriptomic analysis, motile cilia have been identified in the epithelial cells of human endometrium [20,21]. However, the regulation and role of primary cilia during human endometrium remain unclear.

Phosphatase and tensin homologue deleted on chromosome ten (PTEN) is a tumor suppressor gene in a range of human diseases [22,23]. The phosphoinositide 3-kinase (PI3K) mainly catalyzes the production of PtdIns (3,4,5) P_3_. PTEN dephosphorylates PtdIns (3,4,5) P_3_ to form PtdIns (4,5) P_2_ (PIP2) to negatively regulate PI3K/AKT signaling [24,25]. A recent study showed that PIP_2_ determines length and stability of primary cilia by balancing membrane turnovers [26]. Phosphorylated AKT is located at the basal region of the primary cilia [27]. Phosphorylated AKT on serine 473 displays a sharp and significant decrease during human decidualization [28]. PTEN is also involved in multiciliary formation and cilia disassembly [29]. How both AKT and PTEN interact with primary cilium during human decidualization is still unknown.

In this study, we examined primary cilia in human decidual cells and analyzed primary cilia-related mechanism under human decidualization. Our data from both assembly and disassembly of primary cilia indicate that primary cilia are essential to human decidualization. Trichoplein-Aurora A-mediated cilia disassembly is detriment to human decidualization. Primary cilia contribute to human decidualization through PTEN-PI3K-AKT-FOXO1 signaling.

## 2. Results

### 2.1. Primary Cilia Exist in Human Endometrium and during Decidualization

**Figure 1:**
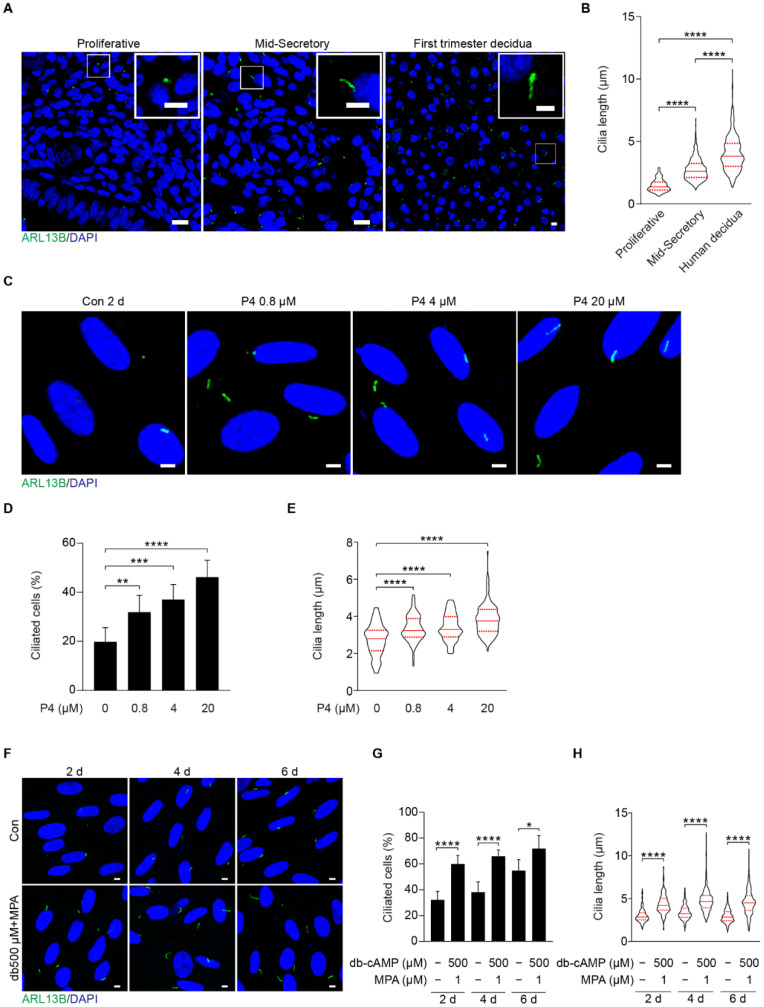
Primary cilia are present in both human endometrium in vivo and in decidual stromal cells in vitro. (**A**) ARL13B immunofluorescence in human endometrium during the proliferative, mid-secretory phase, and human first trimester decidua. ARL13B, a marker of the ciliary membrane, was used to visualize primary cilia (green). Nuclei were stained with DAPI (blue). Scale bars, 10 μm (main image) and 5 μm (magnified regions). (**B**) Cilia length in human endometrium and decidua. (**C**) ARL13B immunofluorescence in progesterone-treated stromal cells for 48 h. Scale bars, 5 μm. (**D**,**E**) Ciliated cells and cilia length were quantified in P_4_-treated stromal cells. (**F**) ARL13B immunofluorescence after stromal cells were induced for decidualization for 2, 4, and 6 days. Scale bars, 5 μm. (**G**,**H**) Ciliated cells and cilia length were quantified after stromal cells were induced for decidualization for 2, 4, and 6 days. In (**A**,**C**,**F**), the representative images of three biologically independent experiments are shown. In (**B**,**E**,**H**), these results are pooled from three independent experiments and presented as violin plots. The thick bar indicates the median and the dotted lines the first and third quartiles. In (**B**,**D**,**E**,**G**,**H**), data are presented as means ± SD from three independent experiments. * *p* < 0.05, ** *p* < 0.01, *** *p* < 0.001, and **** *p* < 0.0001 by two-tailed Student’s *t* test for comparing two groups or one-way ANOVA test for comparing more than two groups.

Because primary cilia are key participants in Hedgehog (HH), WNT, and bone morphogenetic protein (BMP) signaling pathways which are essential to decidualization [13,30], we are wondering whether primary cilium is involved in human decidualization. ARL13B, a marker of the ciliary membrane, was used to recognize primary cilia [31]. Immunofluorescence showed that primary cilia were present in human endometrial stromal cells (Figure 1A). During the menstrual cycle, primary cilia were significantly longer in the secretory phase than those in the proliferative phase (Figure 1A,B). Additionally, primary cilia were much longer in first trimester decidual cells than in menstrual phases (Figure 1B). When human stromal cells were treated with different doses of progesterone, the rate of ciliated cells and cilia length were dose-dependently increased (Figure 1C–E). After human stromal cells were induced for in vitro decidualization for 2, 4, or 6 days, the rate of ciliated cells and cilia length were also significantly increased compared to control groups (Figure 1F–H). These observations indicate that primary cilia are likely involved in human decidualization.

### 2.2. Primary Cilia Are Indispensable for Human Decidualization

**Figure 2:**
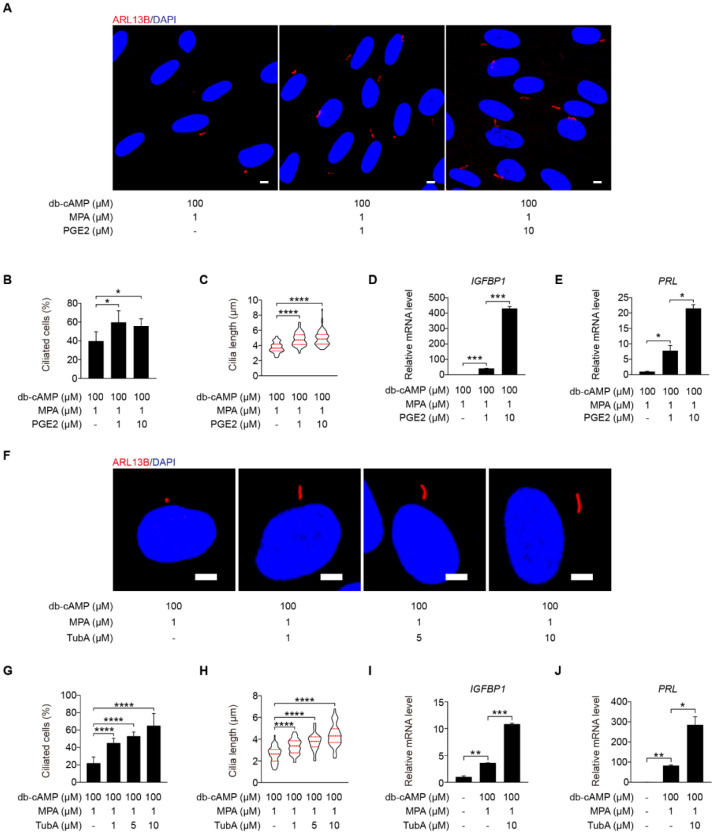
Primary cilia are critical for human decidualization. (**A**) ARL13B immunofluorescence in PGE2-treated stromal cells for 48 h. Scale bars, 5 μm. (**B**,**C**) Ciliated cells and cilia length were measured in PGE2-treated stromal cells for 48 h. (**D**,**E**) Effects of PGE2 treatment on relative mRNA levels of *IGFBP1* and *PRL* under in vitro decidualization of stromal cells for 48 h. (**F**) ARL13B immunofluorescence in TubA-treated stromal cells for 48 h. Scale bars, 5 μm. (**G**,**H**) Ciliated cells and cilia length were measured in TubA-treated stromal cells for 48 h. (**I**,**J**) Relative mRNA levels of *IGFBP1* and *PRL* in TubA-treated stromal cells for 48 h. In (**A**,**F**), the representative images of three biologically independent experiments are shown. In (**C**,**H**), the results are pooled from three independent experiments and presented as violin plots. The thick bar indicates the median and the dotted lines the first and third quartiles. In (**B**–**E**,**G**–**J**), data are presented as means ± SD from three independent experiments. * *p* < 0.05, ** *p* < 0.01, *** *p* < 0.001, and **** *p* < 0.0001 by two-tailed Student’s *t* test for comparing two groups or one-way ANOVA test for comparing more than two groups.

To determine the function of primary cilium during human decidualization, primary cilia were controlled by assembly and disassembly, respectively. Prostaglandin E2 (PGE2) promotes ciliogenesis through stimulating intraflagellar transport [32]. Under in vitro decidualization, PGE2 remarkably stimulated ciliogenesis and cilia length (Figure 2A–C). *IGFBP1* (insulin-like growth factor-binding protein 1) and *PRL* (prolactin), two marker genes of human decidualization [33], were also remarkably enhanced by PGE2 treatment (Figure 2D,E).

**Figure 3:**
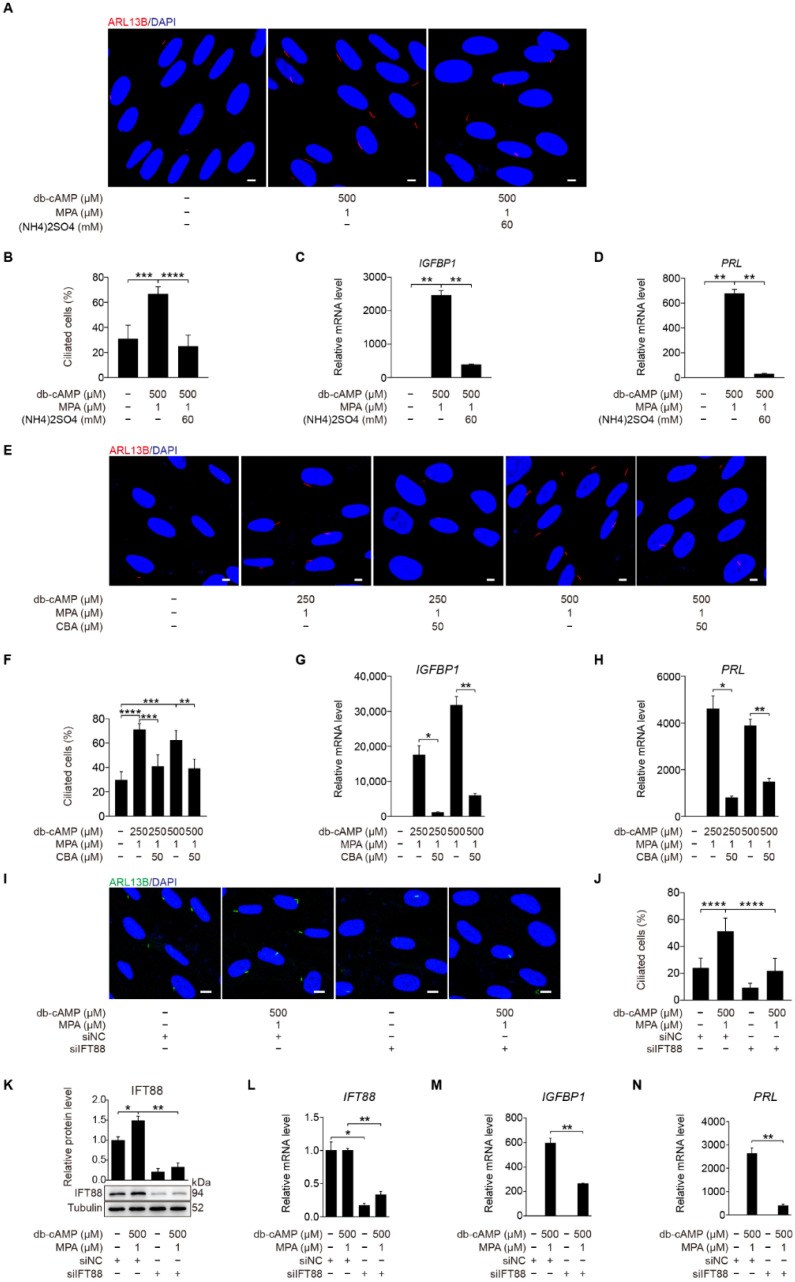
Primary cilia are indispensable for human decidualization. (**A**) ARL13B immunofluorescence in (NH_4_)_2_SO_4_-treated stromal cells for 24 h under in vitro decidualization for 48 h. Scale bars, 5 μm. (**B**) Ciliated cells of stromal cells after (NH_4_)_2_SO_4_ treatment for 24 h under in vitro decidualization for 48 h. (**C**,**D**) Relative mRNA levels of *IGFBP1* and *PRL* of stromal cells after (NH_4_)_2_SO_4_ treatment for 24 h under in vitro decidualization for 48 h. (**E**) Effects of CBA treatment for 24 h on ARL13B immunofluorescence under in vitro decidualization of stromal cells for 48 h. Scale bars, 5 μm. (**F**) Effects of CBA treatment for 24 h on the percentage of ciliated cells under in vitro decidualization of stromal cells for 48 h. (**G**,**H**) Effects of CBA treatment for 24 h on relative mRNA levels of *IGFBP1* and *PRL* under in vitro decidualization of stromal cells for 48 h. (**I**) ARL13B immunofluorescence after stromal cells were transfected with si*IFT88* for 24 h then in vitro decidualization for 48 h. Scale bars, 5 μm. (**J**,**K**) Ciliated cells and IFT88 protein levels after stromal cells were treated with si*IFT88* for 24 h then in vitro decidualization for 48 h. (**L**–**N**) Relative mRNA levels of *IFT88*, *IGFBP1*, and *PRL* after stromal cells were transfected with si*IFT88* for 24 h followed by in vitro decidualization for 48 h. In (**A**,**E**,**I**), the representative images of three biologically independent experiments are shown. In (**B**–**D**,**F**–**H**,**J**–**N**), data are presented as means ± SD from three independent experiments. * *p* < 0.05, ** *p* < 0.01, *** *p* < 0.001, and **** *p* < 0.0001 by two-tailed Student’s *t* test for comparing two groups or one-way ANOVA test for comparing more than two groups.

Tubastatin A (TubA), a selective inhibitor of the deacetylase activity of HDAC6, that is required for cilia disassembly [34]. When stromal cells were treated with TubA under in vitro decidualization, the ciliogenesis and cilia length were significantly enhanced (Figure 2F–H). TubA treatment also induced the mRNA levels of *IGFBP1* and *PRL* (Figure 2I,J).

(NH_4_)_2_SO_4_ is a chemical reagent for deciliation of cells with primary cilia [35]. When stromal cells under in vitro decidualization were treated with (NH_4_)_2_SO_4_ for 24 h, the number of ciliated cells was significantly reduced (Figure 3A,B). The mRNA levels of *IGFBP1* and *PRL* were also remarkably reduced by (NH_4_)_2_SO_4_ treatment (Figure 3C,D).

Ciliobrevin A (CBA) is a specific inhibitor of primary cilia [36]. When stromal cells were treated with CBA under in vitro decidualization, the number of ciliated cells was significantly reduced (Figure 3E,F), followed by remarkably decreased mRNA expression levels of *IGFBP1* and *PRL* (Figure 3G,H). Intraflagellar transport 88 (*IFT88*) is an essential player during ciliogenesis [37]. When stromal cells under in vitro decidualization were treated with IFT88 siRNA, both the number of ciliated cells, and the levels of IFT88 mRNA and protein were significantly decreased (Figure 3I–L). Compared with control siRNA, IFT88 siRNA significantly inhibited the expression of *IGFBP1* and *PRL* mRNA (Figure 3M,N). Taken together, these results imply that primary cilia play a critical role in the regulation of human decidualization.

### 2.3. Aberrant Primary Cilia in the Endometrium of RIF Patients

**Figure 4:**
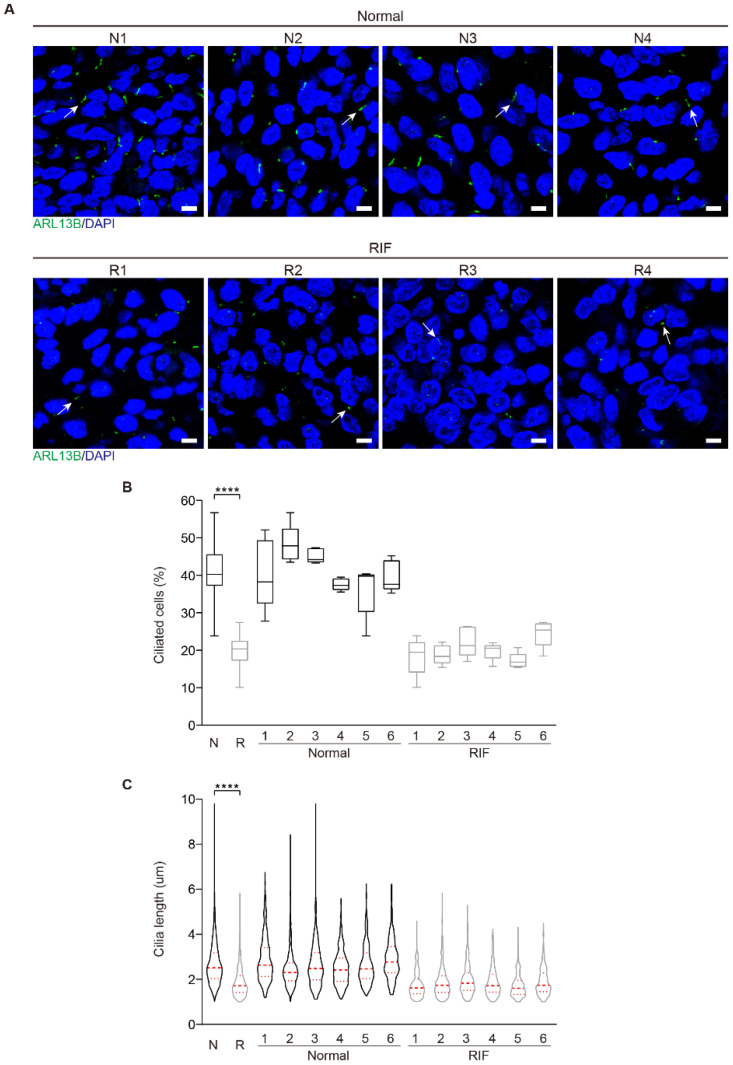
Aberrant primary cilia in the endometrium of RIF patients. (**A**) The representative images of ARL13B immunofluorescence in normal and RIF human endometrium. The representative ARL13B immunofluorescent images were shown from 4 control patients and 4 RIF patients. Primary cilia were identified with ARL13B antibody as green color. Nuclei were stained with DAPI (blue). Arrows, primary cilium; Scale bars, 5 μm. (**B**) Ciliated cells in normal and RIF human endometrium (N = 6, R = 6). The results are presented as median ± min/max whiskers in box plots. (**C**) Cilia length in normal and RIF human endometrium (N = 6, R = 6). The results are presented as violin plots, the thick bar indicates the median and the dotted lines the first and third quartiles. In (**B**,**C**), data are presented as means ± SD. **** *p* < 0.0001 by two-tailed Student’s *t* test for comparing two groups or one-way ANOVA test for comparing more than two groups.

Because impaired decidualization is the dominant cause for RIF [12], we examined primary cilia in the endometrial samples from RIF patients. Immunofluorescence showed that primary cilia were present in human endometrial stromal cells both in normal and RIF patients (Figure 4A). In the endometrium from RIF patients, primary cilia were less and shorter compared with the normal endometrium (Figure 4B,C), implying that primary cilia might be a factor for decidualization failure in RIF endometrium.

### 2.4. Aurora A-Mediated Primary Cilia Disassembly Inhibits Human Decidualization

**Figure 5:**
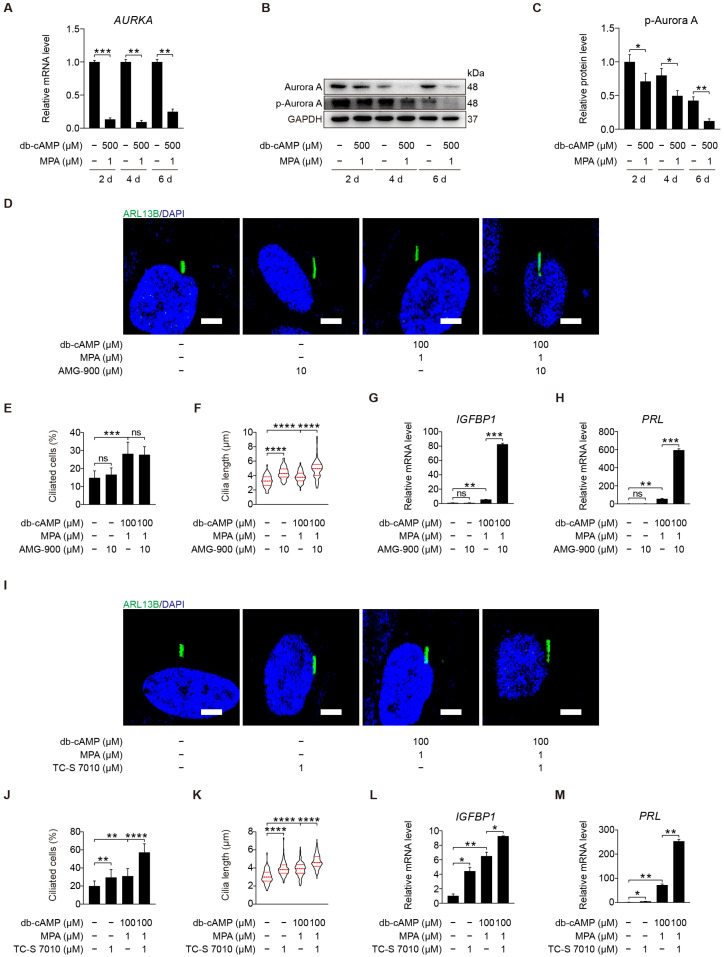
Aurora A-mediated primary cilia disassembly inhibits human decidualization. (**A**) Relative mRNA level of *AURKA* during stromal cells decidualization for 2, 4, and 6 days. (**B**,**C**) Western blot and quantification of p-Aurora A (T288) during stromal cells decidualization for 2, 4, and 6 days. (**D**) ARL13B immunofluorescence in AMG-900-treated stromal cells for 48 h. Scale bars, 5 μm. (**E**,**F**) Ciliated cells and cilia length were measured in AMG-900-treated stromal cells for 48 h. (**G**,**H**) Relative mRNA levels of *IGFBP1* and *PRL* in AMG-900-treated stromal cells for 48 h. (**I**) ARL13B immunofluorescence in TC-S 7010-treated stromal cells for 48 h. Scale bars, 5 μm. (**J**,**K**) Ciliated cells and cilia length were measured in TC-S 7010-treated stromal cells for 48 h. (**L**,**M**) Relative mRNA levels of *IGFBP1* and *PRL* in TC-S 7010-treated stromal cells for 48 h. In (**B**,**D**,**I**), the representative images of three biologically independent experiments are shown. In (**F**,**K**), these results are pooled from three independent experiments and presented as violin plots. The thick bar indicates the median and the dotted lines the first and third quartiles. In (**A**,**C**,**E**–**H**,**J**–**M**), data are presented as means ± SD from three independent experiments. ns, (*p* > 0.05), * *p* < 0.05, ** *p* < 0.01, *** *p* < 0.001, and **** *p* < 0.0001 by two-tailed Student’s *t* test for comparing two groups or one-way ANOVA test for comparing more than two groups.

Aurora A activation at basal body can induce cilia disassembly and is a prerequisite for mitosis [17,38]. It is unclear whether Aurora A is involved in human decidualization through cilia disassembly. Aurora kinase A (*AURKA*) mRNA level was dramatically mitigated after human stromal cells were induced for in vitro decidualization for 2, 4, or 6 days (Figure 5A). Aurora A is activated by phosphorylation at Thr288 [39]. Western blot showed that the protein levels of Aurora A and p-Aurora A (T288) were significantly decreased after human stromal cells were induced for in vitro decidualization for 2, 4, or 6 days (Figure 5B,C and Figure S1A). Under in vitro decidualization, AMG-900, a pan-Aurora kinase inhibitor, had little effects on ciliogenesis, but significantly increased ciliary length (Figure 5D–F). AMG-900 treatment significantly stimulated the mRNA levels of *IGFBP1* and *PRL* (Figure 5G,H). When stromal cells under in vitro decidualization were treated with TC-S 7010, a specific inhibitor of Aurora A, both ciliogenesis and cilia length were obviously increased (Figure 5I–K). TC-S 7010 treatment also significantly induced the mRNA levels of *IGFBP1* and *PRL* (Figure 5L,M). These results demonstrated that Aurora A could suppress human in vitro decidualization through cilia disassembly.

### 2.5. Trichoplein Acts as the Activator of Aurora A

**Figure 6:**
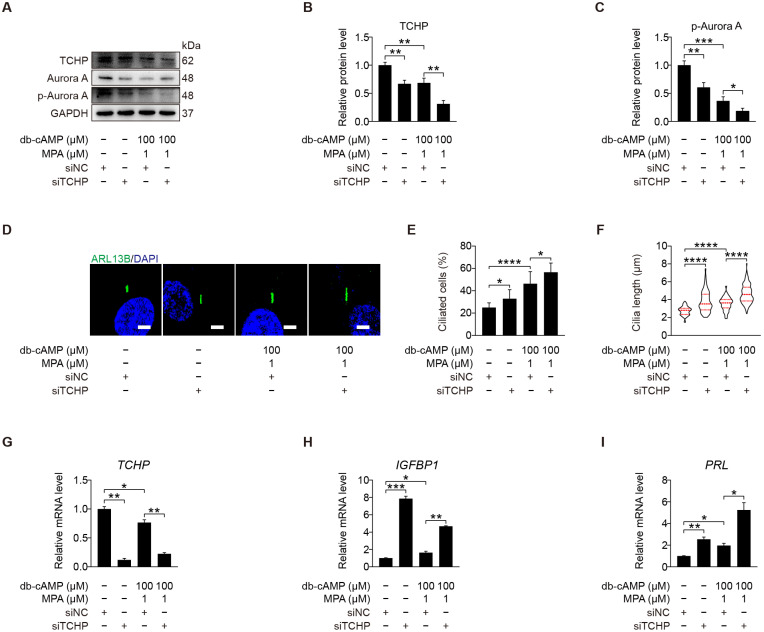
Trichoplein acts as the activator of Aurora A. (**A**–**C**) Western blot and quantification of TCHP and p-Aurora A (T288) after stromal cells were transfected with siTCHP for 24 h then in vitro decidualization for 48 h. (**D**) ARL13B immunofluorescence after stromal cells were transfected with siTCHP for 24 h then in vitro decidualization for 48 h. Scale bars, 5 μm. (**E**,**F**) Ciliated cells and cilia length were analyzed after stromal cells were transfected with siTCHP for 24 h then in vitro decidualization for 48 h. (**G**–**I**) Relative mRNA levels of *TCHP*, *IGFBP1*, and *PRL* after stromal cells were transfected with si*TCHP* for 24 h then in vitro decidualization for 48 h. In (**A**,**D**), the representative images of three biologically independent experiments are shown. In (**F**), the results are pooled from three independent experiments and presented as violin plots. The thick bar indicates the median and the dotted lines the first and third quartiles. In (**B**,**C**,**E**–**I**), data are presented as means ± SD from three independent experiments. * *p* < 0.05, ** *p* < 0.01, *** *p* < 0.001, and **** *p* < 0.0001 by two-tailed Student’s *t* test for comparing two groups or one-way ANOVA test for comparing more than two groups.

We demonstrated that kinase Aurora A acts as a negative regulator of primary cilia during human decidualization. Trichoplein is localized at mother and daughter centrioles in proliferating cells and identified as the activator of Aurora A to disassemble primary cilia [40]. We were wondering whether TCHP is involved in human decidualization through cilia disassembly. When stromal cells under in vitro decidualization were treated with TCHP siRNA, the level of p-Aurora A (T288) protein was significantly alleviated (Figure 6A–C and Figure S2A), followed by an obvious increase in ciliated cells and cilia length (Figure 6D–F). Treatment with TCHP siRNA also caused a significant increase in the mRNA levels of *IGFBP1* and *PRL* (Figure 6G–I). These results suggest that trichoplein could act as the upstream of Aurora A to negatively modulate primary cilia during human decidualization.

### 2.6. Primary Cilia Negatively Control AKT Activation

**Figure 7:**
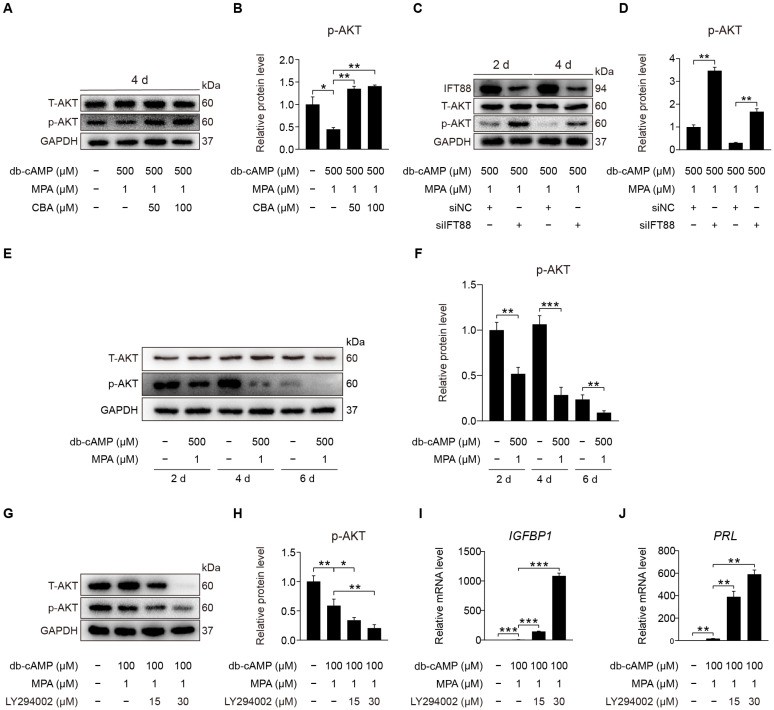
Primary cilia negatively control AKT activation. (**A**,**B**) Western blot and quantification of p-AKT (S473) after stromal cells were treated with CBA for 24 h during in vitro decidualization for 4 days. (**C**,**D**) Western blot and quantification of p-AKT (S473) after stromal cells were treated with siIFT88 for 24 h then in vitro decidualization for 2 and 4 days. (**E**,**F**) Western blot and quantification of p-AKT (S473) in stromal cells during decidualization for 2, 4, and 6 days. (**G**,**H**) Western blot and quantification of p-AKT (S473) in LY294002-treated stromal cells for 48 h. (**I**,**J**) Relative mRNA levels of *IGFBP1* and *PRL* in LY294002-treated stromal cells for 48 h. In (**A**,**C**,**E**,**G**), the representative images of three biologically independent experiments are shown. In (**B**,**D**,**F**,**H**–**J**), data are presented as means ± SD from three independent experiments. ns, (*p* > 0.05), * *p* < 0.05, ** *p* < 0.01, and *** *p* < 0.001 by two-tailed Student’s *t* test for comparing two groups or one-way ANOVA test for comparing more than two groups.

After primary cilia were shown to be involved in promoting human decidualization, we further investigated the downstream signaling pathways through which primary cilia might mediate human decidualization. Phosphorylated AKT was located at the basal region of the primary cilia [27]. We would like to examine whether primary cilia modulate human decidualization via PI3K-AKT signaling. When stromal cells were treated with CBA under in vitro decidualization, the level of AKT phosphorylation was significantly increased under in vitro decidualization for 2 or 4 days (Figure 7A,B and Figure S3A–D). After stromal cells were treated with IFT88 siRNA under in vitro decidualization, IFT88 protein level was significantly decreased (Figure S3E), followed by the increased protein level of p-AKT (S473) (Figure 7C,D and Figure S3F). Western blot showed that the protein level of AKT phosphorylation at S473 was significantly decreased after stromal cells were induced for in vitro decidualization for 2, 4, or 6 days (Figure 7E,F and Figure S3G). When stromal cells under in vitro decidualization were treated with LY294002, the inhibitor of phosphoinositide 3-kinase (PI3K)-AKT pathway, p-AKT (S473) protein level was obviously suppressed (Figure 7G,H and Figure S3H), followed by a significant increase in *IGFBP1* and *PRL* mRNA levels (Figure 7I,J). In short, primary cilia mainly serve as the suppressor of PI3K-AKT signaling during human decidualization.

### 2.7. PTEN Antagonizes AKT during Human Decidualization

**Figure 8:**
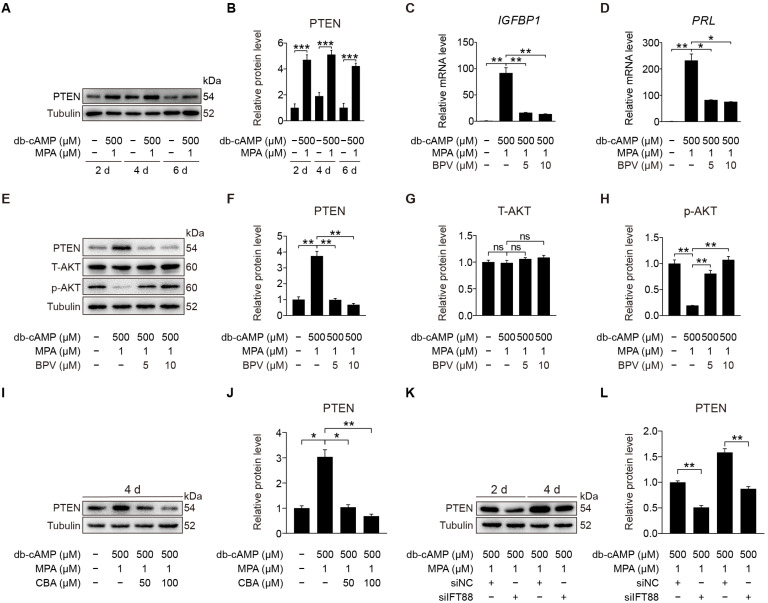
PTEN antagonizes AKT during stromal cells decidualization. (**A**,**B**) Western blot and quantification of PTEN in stromal cells during decidualization for 2, 4, and 6 days. (**C**,**D**) Relative mRNA levels of *IGFBP1* and *PRL* in BPV-treated stromal cells for 48 h. (**E**–**H**) Western blot and quantification of PTEN, T-AKT, and p-AKT (S473) in BPV-treated stromal cells for 48 h. (**I**,**J**) Western blot and quantification of PTEN after stromal cells were treated with CBA for 24 h during in vitro decidualization for 4 days. (**K**,**L**) Western blot and quantification of PTEN after stromal cells were treated with siIFT88 for 24 h then in vitro decidualization for 2 and 4 days. In (**A**,**E**,**I**,**K**), the representative images of three biologically independent experiments are shown. In (**B**–**D**,**F**–**H**,**J**–**L**), data are presented as means ± SD from three independent experiments. ns, (*p* > 0.05), * *p* < 0.05, ** *p* < 0.01, and *** *p* < 0.001 by two-tailed Student’s *t* test for comparing two groups or one-way ANOVA test for comparing more than two groups.

PTEN is the main negative regulator of the PI3K pathway and participates in multi-cilia formation and cilia disassembly [29,41]. Primary cilia loss causes destabilization of PTEN and activation of AKT [42]. We would like to see whether primary cilia modulate human decidualization through PTEN. Western blot displayed that PTEN was dramatically promoted after stromal cells were induced for in vitro decidualization for 2, 4, or 6 days (Figure 8A,B). When stromal cells under in vitro decidualization were treated with BPV, a specific inhibitor for PTEN [43], the mRNA levels of *IGFBP1* and *PRL* were significantly suppressed (Figure 8C,D). BPV treatment also effectively decreased the protein levels of PTEN and increased p-AKT (S473) protein levels (Figure 8E–H). When stromal cells under in vitro decidualization were treated with CBA, PTEN protein level was significantly decreased (Figure 8I,J and Figure S4A,B). Similarly, IFT88 knockdown also remarkably reduced PTEN protein level (Figure 8K,L). These suggest that primary cilia may regulate human decidualization through positive regulating PTEN.

### 2.8. Primary Cilia Modulate FOXO1 Expression by Suppressing AKT Activity

**Figure 9:**
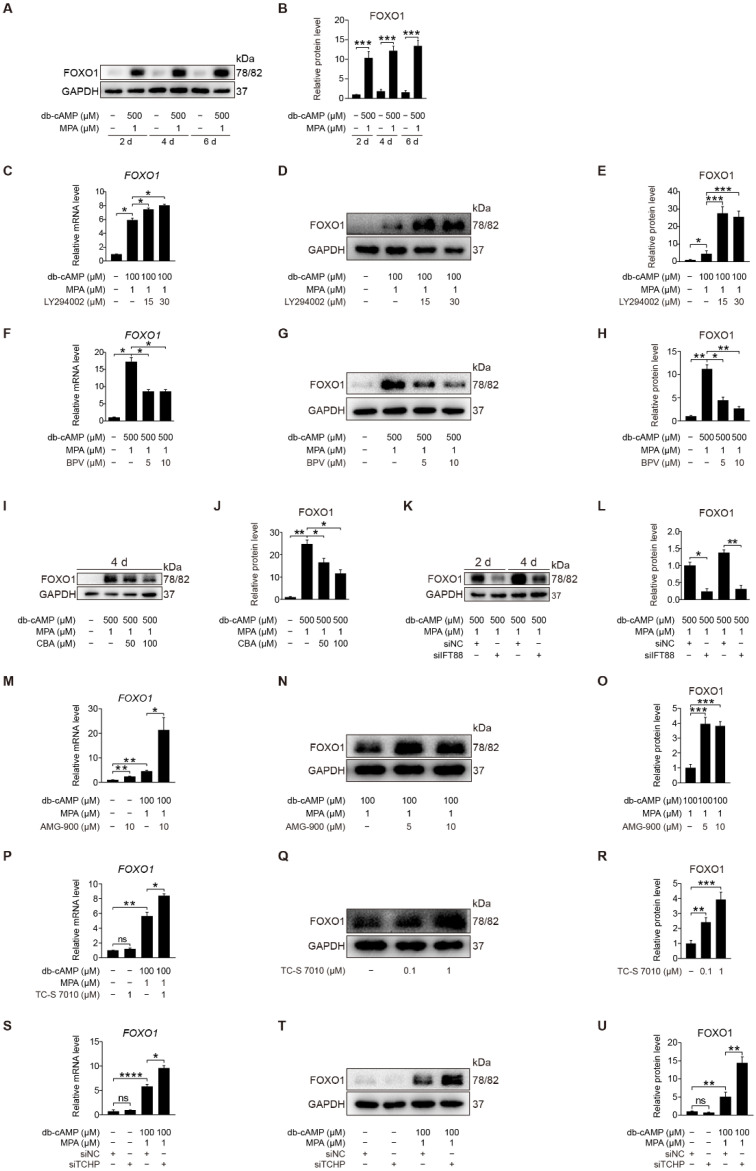
Primary cilia modulate FOXO1 expression by suppressing AKT activity. (**A**,**B**) Western blot and quantification of FOXO1 in stromal cells during decidualization for 2, 4, and 6 days. (**C**) Relative mRNA level of *FOXO1* in LY294002-treated stromal cells for 48 h. (**D**,**E**) Western blot and quantification of FOXO1 in LY294002-treated stromal cells for 48 h. (**F**) Relative mRNA level of *FOXO1* in BPV-treated stromal cells for 48 h. (**G**,**H**) Western blot and quantification of FOXO1 in BPV-treated stromal cells for 48 h. (**I**,**J**) Western blot and quantification of FOXO1 after stromal cells were treated with CBA for 24 h during in vitro decidualization for 4 days. (**K**,**L**) Western blot and quantification of FOXO1 after stromal cells were treated with siIFT88 for 24 h then in vitro decidualization for 2 and 4 days. (**M**) Relative mRNA level of *FOXO1* in AMG-900-treated stromal cells for 48 h. (**N**,**O**) Western blot and quantification of FOXO1 in AMG-900-treated stromal cells for 48 h. (**P**) Relative mRNA level of *FOXO1* in TC-S 7010-treated stromal cells for 48 h. (**Q**,**R**) Western blot and quantification of FOXO1 in TC-S 7010-treated stromal cells for 48 h. (**S**) Relative mRNA level of *FOXO1* after stromal cells were treated with siTCHP for 24 h then in vitro decidualization for 2 days. (**T**,**U**) Western blot and quantification of FOXO1 after stromal cells were treated with siTCHP for 24 h then in vitro decidualization for 2 days. In (**A**,**D**,**G**,**I**,**K**,**N**,**Q**,**T**), the representative images of three biologically independent experiments are shown. In (**B**,**C**,**E**,**F**,**H**,**M**,**J**,**L**,**M**,**O**,**P**,**R**,**S**,**U**), data are presented as means ± SD from three independent experiments. ns, (*p* > 0.05), * *p* < 0.05, ** *p* < 0.01, *** *p* < 0.001 and **** *p* < 0.0001 by two-tailed Student’s *t* test for comparing two groups or one-way ANOVA test for comparing more than two groups.

FOXO1 is a direct phosphorylation target of AKT [44]. During human decidualization, FOXO1 is distinctly increased [45]. We were wondering whether FOXO1 is involved in primary cilium regulation on human decidualization. When stromal cells were induced for in vitro decidualization for 2, 4, or 6 days, FOXO1 protein level was dramatically increased (Figure 9A,B). When stromal cells under in vitro decidualization were treated with LY294002, a specific inhibitor of PI3K/AKT, FOXO1 mRNA and protein levels were significantly stimulated (Figure 9C–E). Treatment of stromal cells with BPV significantly decreased FOXO1 mRNA and protein levels (Figure 9F–H). When stromal cells under in vitro decidualization for 2 or 4 days were treated with either CBA or IFT88 siRNA, FOXO1 protein level was remarkably alleviated (Figure 9I–L and Figure S5A,B). To see whether Aurora A has a regulation on FOXO1, stromal cells under in vitro decidualization were treated with Aurora A inhibitors. Treatment with AMG-900 or TC-S 7010 significantly stimulated both FOXO1 mRNA and protein levels (Figure 9M–R). Treatment with TCHP siRNA also significantly increased FOXO1 mRNA and protein levels (Figure 9S–U). Collectively, these results suggest that primary cilia should modulate human decidualization through PTEN-AKT-FOXO1 pathway.

### 2.9. Trichoplein-Aurora A-Mediated Cilia Disassembly Interacts with PTEN-AKT-FOXO1 Signaling

**Figure 10:**
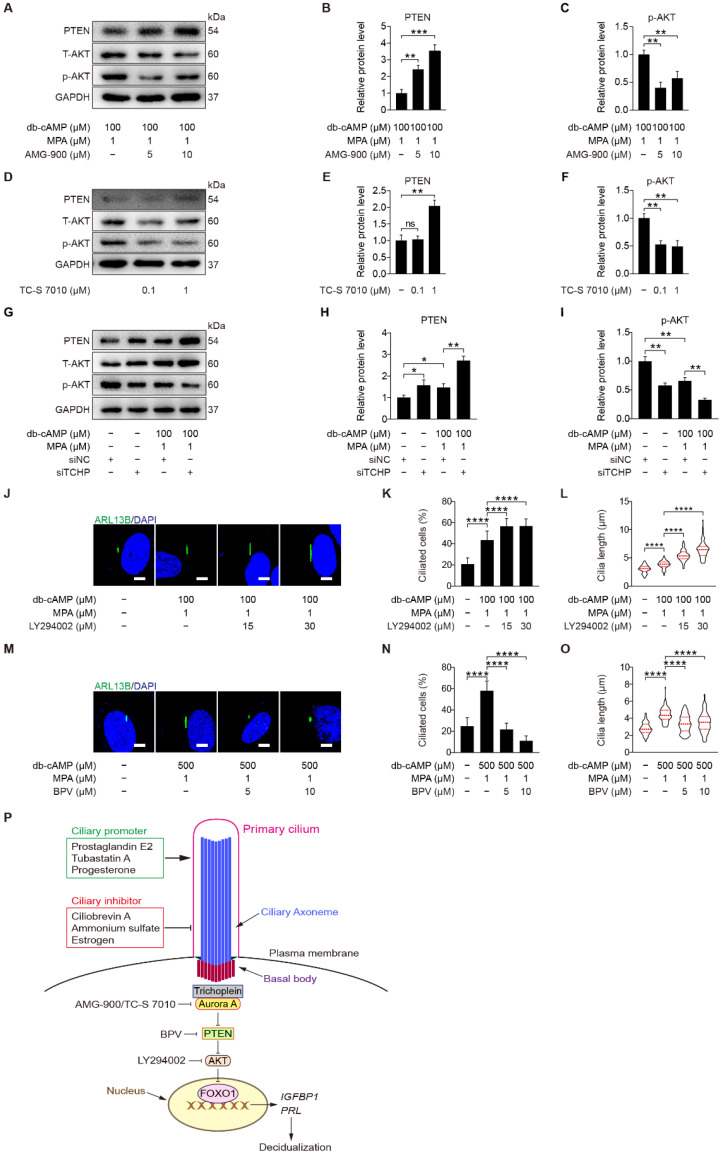
Trichoplein-Aurora A-mediated cilia disassembly contributes to PTEN-AKT-FOXO1 signaling. (**A**–**C**) Western blot and quantification of PTEN and p-AKT in AMG-900-treated stromal cells for 48 h. (**D**–**F**) Western blot and quantification of PTEN and p-AKT in TC-S 7010-treated stromal cells for 48 h. (**G**–**I**) Western blot and quantification of PTEN and p-AKT after stromal cells were transfected with siTCHP for 24 h then in vitro decidualization for 48 h. (**J**) ARL13B immunofluorescence in LY294002-treated stromal cells for 48 h. Scale bars, 5 μm. (**K**,**L**) Ciliated cells and cilia length were measured in LY294002-treated stromal cells for 48 h. (**M**) ARL13B immunofluorescence in BPV-treated stromal cells for 48 h. Scale bars, 5 μm. (**N**,**O**) Ciliated cells and cilia length were measured in BPV-treated stromal cells for 48 h. (**P**) Model of primary cilia function in stromal cells decidualization. In (**A**,**D**,**G**,**J**,**M**), the representative images of three biologically independent experiments are shown. In (**L**,**O**), the results are pooled from three independent experiments and presented as violin plots. The thick bar indicates the median and the dotted lines in the first and third quartiles. In (**B**,**C**,**E**,**F**,**H**,**I**,**K**,**L**,**N**,**O**), data are presented as means ± SD from three independent experiments. ns, (*p* > 0.05), * *p* < 0.05, ** *p* < 0.01, *** *p* < 0.001, and **** *p* < 0.0001 by two-tailed Student’s *t* test for comparing two groups or one-way ANOVA test for comparing more than two groups.

Both AURKA and AKT are located at the ciliary base [46]. In ovarian cancer cells, Aurora A motivates AKT through suppressing PTEN [47]. We were wondering whether Aurora A could regulate PTEN and AKT during human decidualization. When stromal cells under in vitro decidualization were treated with AMG-900 or TC-S 7010, there were a significant increase of PTEN and a significant decrease of p-AKT (S473) protein level (Figure 10A–F and Figure S6A,B). Because depletion of either trichoplein or Aurora A limits IGF1R-AKT signaling [48], we checked the effect of trichoplein on PI3K-AKT signaling. After stromal cells under in vitro decidualization were treated with TCHP siRNA, there was an increase in PTEN protein level and a decrease in AKT phosphorylation (Figure 10G–I and Figure S6C). These results demonstrate that TCHP-Aurora A-mediated cilia disassembly is necessary for AKT activation. On the other hand, we found that inhibition of AKT in stromal cells led to increased ciliated cells and cilia length (Figure 10J–L), while inhibition of PTEN caused decreased ciliated cells and cilia length (Figure 10M–O). These results suggest that PTEN-AKT might also have a feedback effect on primary cilia, although TCHP-Aurora A mainly modulates PTEN-AKT via primary cilia during human decidualization (Figure 10P).

## 3. Discussion

In this study, we showed that primary cilia are present in human endometrial stromal cells and longer in decidual cells than in stromal cells during a menstrual cycle. The length and ciliogenesis of primary cilia were stimulated by progesterone and under in vitro decidualization. Inhibition of primary cilia caused impaired human decidualization. Abnormal primary cilia are detected in RIF patients. Our data suggests that primary cilia modulates human decidualization through trichoplein-Aurora A-PI3K-AKT signaling.

Progesterone and estrogen are two essential prerequisites for endometrial decidualization in both mice and humans [49]. In our study, progesterone significantly increases the rate of ciliated cells and cilia length in stromal cells of endometrial samples and cultured stromal cells. In oviducts, estrogen drives multi-ciliogenesis through estrogen receptor β [50]. In human endometrial organoids, ciliated cells are governed by estrogen and Notch signaling. Additionally, estrogen serves as the primitive driver for ciliogenesis [51]. A recent study showed that cilia-related gene expression was significantly altered in aged endometrium [52]. Ciliated cells are present in human endometrial epithelium [21]. It seems that progesterone has a stimulatory effect on primary cilia in stromal cells, while estrogen shows a promoting effect on the cilia in endometrial epithelium.

Our data from both assembly and disassembly of primary cilia indicate that primary cilia are essential to human decidualization. Primary cilia are nucleated by basal bodies and mainly present in non-dividing cells including quiescent (G0) and differentiated cells [53]. PGE2 stimulates ciliogenesis [32], and is also able to induce human decidualization [54]. HDAC6 is required for cilia disassembly [17]. Tubastatin A (TubA), a selective inhibitor of HDAC6, can stimulate ciliogenesis and human decidualization. IFT88 is important for cilia assembly and maintenance [15]. The inhibition of primary cilia by (NH_4_)_2_SO_4_, CBA, and IFT88 siRNA leads to suppression of human decidualization. The length of primary cilia has a direct impact on cell cycle reentry. Longer primary cilia need to take more time for disassembly, and short cilia should quickly meet the cell cycle [55]. In our study, progesterone obviously stimulates ciliogenesis of primary cilia. Progesterone is essential to human endometrial receptivity and decidualization [56,57]. Progesterone-targeted Ihh, Wnt4, and BMP2 signaling plays a key role during mouse and human decidualization [56]. It seems that longer primary cilia may be a checkpoint for human decidualization.

Aurora A is localized at basal body of primary cilia, and Aurora A phosphorylation at T288 can induce cilia disassembly [17,38]. Trichoplein is co-localized with Aurora A in primary cilia and can activate Aurora A for disassembling primary cilia [40]. In our study, treatment of stromal cells with Aurora A inhibitors or TCHP siRNA both can promote ciliogenesis and human decidualization, also confirming that primary cilia should be required for human decidualization.

Primary cilia act as chemosensory function for tumor suppression through PTEN-AKT-dependent mechanism in cholangiocytes [42]. AKT is localized in basal region of primary cilia [27]. Loss of primary cilia cause AKT activation [58]. A previous study showed that PI3K-AKT signaling is negatively modulated during human decidualization [28]. In our study, primary cilia negatively control PI3K-AKT pathway. AURKA and AKT are co-localized in primary cilia [46]. Inhibition of Aurora A in stromal cells also down-regulates AKT phosphorylation in our study. PTEN is a negative regulator of AKT activation [29,41]. In human endometrium, PTEN protein level is increased by progesterone [59]. Our results also showed that PTEN is remarkably stimulated by MPA and cAMP. Human decidualization is impaired by inhibiting PTEN. Additionally, disassembly of primary cilia suppresses PTEN level. FOXO1 transcriptionally controls *IGFBP1* and *PRL* expression [60]. PTEN can activate FOXO1 and inhibit AKT phosphorylation [61,62]. Our study showed defective primary cilia in the endometrial cells from RIF patients. Decidualization abnormality may contribute to recurrent implantation failure [11]. In RIF endometrial samples, there is an increase of AKT activation and a decrease in FOXO1 expression [63]. Our data shows that primary cilia are important for FOXO1 activation during human decidualization.

In summary, primary cilia are present in human decidua and should be important for human decidualization. Primary cilia modulate human decidualization mainly through TCHP-Aurora A-PI3K-AKT signaling.

## 4. Materials and Methods

### 4.1. Collection of Human Endometrial Samples

Human endometrial samples during menstrual cycle were collected by biopsy from normally cycling women 25–40 years old with informed consent. Endometrial samples were dated for menstrual cycle phase according to endometrial morphology and menstrual history [64]. Decidual tissues were obtained from women 31–38 years old undergoing elective terminations of first-trimester pregnancy. In total, six endometrial biopsies for each menstrual cycle phase and six decidua tissues were used in this study. All human procedures regarding endometrial biopsies and decidual tissues were approved by Ethical Committee of Women’s Hospital, School of Medicine, Zhejiang University.

The endometrial biopsies used for RIF study were obtained from all patients with written informed consent. Mid-secretory endometria timed 6–8 days after exogenous progesterone treatment in the non-transfer cycle were obtained from six normal women and six women with RIF undergoing IVF-ET. The normal group was composed of women whose infertility was due to male factors and were confirmed to be fertile after their first IVF-ET treatment. RIF was defined as failure to achieve pregnancy after the transfer of at least four good-quality cleavage-stage embryos or no less than two good-quality blastocysts over a minimum of two consecutive fresh or frozen cycles as described previously [65]. These groups showed no difference in age, body mass index, or menstrual cycles. Patients with endometriosis, adenomyosis, endometrial hyperplasia, endometrial polyps, polycystic ovarian syndrome (PCOS), or hydrosalpinx were excluded.

### 4.2. Culture and Treatments of Human Endometrial Stromal Cells

The immortalized human endometrial stromal cell line (ATCC, CRL-4003^TM^) cells were cultured as previously described [66]. Human endometrial stromal cells were seeded in 12-well plates and cultured with DMEM/F12 (Merck) containing 10% cFBS (Biological Industries) until ~80% confluency at 37 °C and 5% CO_2_. In vitro decidualization was performed as previously described [67]. Briefly, human endometrial stromal cells were induced by 100 or 500 μM dibutyryl cyclic adenosine monophosphate (db-cAMP, D0627, Merck) and 1 μM medroxyprogesterone acetate (MPA, M1629, Merck) for indicated times. Insulin-like growth factor binding protein-1 (*IGFBP1*) and prolactin (*PRL*) are reliable markers for evaluating human in vitro decidualization [67]. The reagents used in this study included Tubastatin A (TubA, S8049, Selleck Chemicals, Houston, TX, USA), ciliobrevin A (CBA, S8249, Selleck Chemicals), ammonium sulfate ((NH_4_)_2_SO_4_, A610060, Sangon, China), BPV (HOpic) (S8651, Selleck Chemicals), LY294002 (S1105, Selleck Chemicals), AMG-900 (S2719, Selleck Chemicals), TC-S 7010 (S1451, Selleck Chemicals), and prostaglandin E_2_ (PGE2, 14010, Cayman chemical).

### 4.3. Transfection of Small Interfering RNA

The small interfering RNA (siRNA) and scrambled negative control (NC) siRNA were designed and synthesized by Ribobio Co, Ltd. (Guangzhou, China) with sequences as follows: siRNA-human-*IFT88*: 5′-CGAAGTTCTTTACCAGATA-3′, siRNA-human-*TCHP*: 5′-CAGGCAGAATGGAGCTCTA-3′. Human endometrial stromal cells were transfected with siRNAs as per the manufacturer’s instructions (Lipofectamine 2000 kit, 11668019, Invitrogen, Carlsbad, CA, USA).

### 4.4. Real-Time PCR

RNA extraction and real-time PCR procedures were performed as previously described [68]. Total RNAs of cultured stromal cells were extracted using AG RNAex Pro Reagent (Accurate Biotechnology, Hunan, China) and genomic DNA was digested with RQ1 deoxyribonuclease I (Promega) to purify RNA. Next, 500 ng of RNA from each sample was reverse transcribed into cDNA with HiScript II Q RT SuperMix for qPCR (Vazyme Biotech Co., Ltd., Nanjing, China) according to the manufacturer’s instructions. Real-time PCR was executed with an SYBR Premix Ex Taq kit (Q311-02-AA, Vazyme, China) on the CFX96 Touch Real-Time System (Bio-Rad, Hercules, CA, USA). Relative mRNA fold changes were standardized to a housekeeping gene (*RPL7* in humans) by the 2^−ΔΔCt^ method. All primer sequences used for real-time PCR were listed in Table 1.

### 4.5. Western Blot

Western blot was carried out as previously stated [69]. Briefly, cultured cells were lysed in lysis buffer (50 mM Tris-HCl, pH 7.5; 150 mM NaCl; 0.25% sodium deoxycholate and 1% Triton X-100). The protein concentrations of lysate samples were assessed by BCA kit (Thermo Fisher Scientific, Waltham, MA, USA). Lysate samples were separated by 12% SDS/PAGE gels and transferred onto PVDF membranes (Merck KGaA, Darmstadt, Germany). After blocked with 5% non-fat milk (A600669, Sangon, China) for 1 h, membranes were incubated with each primary antibody overnight at 4 °C and with horseradish peroxidase (HRP)-conjugated secondary antibody (1:5000, Invitrogen) for 1 h. Signals were visualized by ECL substrate (WBKLS0100, Millipore, Billerica, MA, USA) and detected with Tanon Imaging System (5200, Tanon, Shanghai, China). The primary antibodies used were as follows: rabbit anti-IFT88 antibody (1:2000, 13967-1-AP, Proteintech), rabbit anti-PTEN (1:1000, 9188s, Cell Signaling), rabbit anti- p-AKT (ser473) (1:1000, 4058s, Cell Signaling), rabbit anti-T-AKT (1:1000, 9272s, Cell Signaling), rabbit anti-FOXO1 antibody (1:1000, 2880s, Cell Signaling), rabbit anti-Phospho-Stat3 (Tyr705) (1:1000, 9131s, Cell Signaling), mouse anti-Stat3 antibody (1:1000, 9139s, Cell Signaling), rabbit anti- Aurora A (1:1000, 14475s, Cell Signaling), rabbit anti-Phospho-Aurora A (Thr288) Antibody (1:1000, AF3011, Affinity Biosciences), rabbit anti-TCHP antibody (1:1000, 25931-1-AP, Proteintech), mouse anti-GAPDH antibody (1:1000, SC-32233, Santa Cruz), and rabbit anti-α-Tubulin antibody (1:1000, 2144s, Cell Signaling).

### 4.6. Immunofluorescence

Immunofluorescence was performed with modifications according to a previous study [70]. In brief, the uterine paraffin sections were dewaxed and dehydrated. Cultured cells seeded on sterile coverglasses in 24-well plates were fixed with 4% paraformaldehyde in PBS for 30 min and permeabilized with 0.1% Triton X-100 in PBS for 15 min. Then the paraffin sections or cultured cells on coverglasses were blocked with 5% BSA for 1 h at 37 °C and incubated with rabbit anti-ARL13B antibody (1:2000, 17711-1-AP, Proteintech) overnight at 4 °C in a humid chamber. Sections were then incubated with Alexa Fluor^®^ 488-conjugated second antibody (111-545-144, Jackson ImmunoResearch Laboratories, West Grove, PA, USA) or Alexa Fluor^®^ 594-conjugated second antibody (111-585-144, Jackson ImmunoResearch Laboratories). Nuclei were counterstained with DAPI (D9542, Merck). Images were acquired with Leica TCS SP8 scanning laser confocal microscope. For analyzing primary cilia, ciliated cells are countered by Image J (NIH), and cilia length was analyzed manually by Imaris (version 7.4.2, Bitplane) with the measurement tools.

### 4.7. Statistics Analysis

Data were presented as the mean ± standard deviation (SD) unless otherwise specified. The difference between groups was evaluated by one-way ANOVA followed by a two-tailed unpaired Student’s t-test. *p* < 0.05 was considered as statistical significance. Post-hoc power analysis was performed by using G*Power. The powers of all the human data in this study were more than 0.95.

## Figures and Tables

**Table 1:** Primer sequences used in this study.

Genes	Primers
*AURKA-Homo-F*	5′-AGGAGGAACTGGCATCAA-3′
*AURKA-Homo-R*	5′-TTAGGTAGACTCTGGTAGCA-3′
*FOXO1-Homo-F*	5′-CGAGCTGCCAAGAAGAAA-3′
*FOXO1-Homo-R*	5′-TTCGAGGGCGAAATGTAC-3′
*IFT88-Homo-F*	5′-GTTATGATTGGTGCGTGGAAGT-3′
*IFT88-Homo-R*	5′-GGGCTGAGAGATTGGTTGCAG-3′
*IGFBP1-Homo-F*	5′-CCAAACTGCAACAAGAATG-3′
*IGFBP1-Homo-R*	5′-GTAGACGCACCAGCAGAG-3′
*PRL-Homo-F*	5′-AAGCTGTAGAGATTGAGGAGCAAA-3′
*PRL-Homo-R*	5′-TCAGGATGAACCTGGCTGACTA-3′
*RPL7-Homo-F*	5′-CTGCTGTGCCAGAAACCCTT-3′
*RPL7-Homo-R*	5′-TCTTGCCATCCTCGCCAT-3′
*TCHP-Homo-F*	5′-AAGAAGGAGCAGGAGAATC-3′
*TCHP-Homo-R*	5′-TGAGCGTTATACTGATGTCT-3′

## Data Availability

All the data generated in this study are included in this manuscript.

## Supplementary Materials

The following supporting information can be downloaded at: https://www.mdpi.com/article/10.3390/ijms232415573/s1.

## Abbreviations

AURKA	Aurora kinase A
BMP	Bone morphogenetic protein
CBA	Ciliobrevin A
HH	Hedgehog
IFT88	Intraflagellar transport 88
IGFBP1	Insulin-like growth factor-binding protein 1
PGE2	Prostaglandin E2
PI3K	Phosphoinositide 3-kinase
PRL	Prolactin
PTEN	Phosphatase and tensin homologue deleted on chromosome ten
RIF	recurrent implantation failure
TCHP	Trichoplein
TubA	Tubastatin A
