# Supplementary material for: Primary Cilia Restrain PI3K-AKT Signaling to Orchestrate Human Decidualization
Source: Int J Mol Sci. 2022 Dec 8;23(24):15573. doi: 10.3390/ijms232415573 (PMC9779442; doi:10.3390/ijms232415573)
Supplement: Supplementary file 1 [file ijms-23-15573-s001.zip › ijms-2035090-supplementary.pdf]

International Journal of *Molecular Sciences*

MDPI

Primary Cilia Restrain PI3K-AKT Signaling to Orchestrate

Human Decidualization

Bo Li[1,2], Ya-Ping Yan[1], Chen Liang[1], Yu-Ying He[1], Ying Wang[1], Meng-Yuan Li[1], Si-Ting Chen[1], Yue Li[1], Ai-Xia Liu[3], Gui-Jun Yan[4]* and Zeng-Ming Yang[1,2]*

Supplemental materials

A

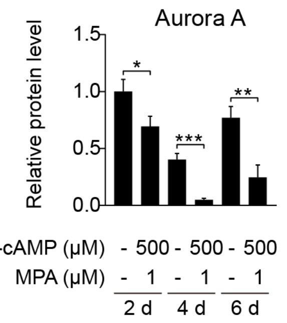

Figure S1. Aurora A-mediated primary cilia disassembly inhibits human decidualization. **(A)** Western blot quantification of Aurora A during stromal cells decidualization for 2, 4, and 6 days. In (**A**), data are presented as means ± SD from three independent experiments. * P<0.05, ** P<0.01, and *** P<0.001 by two-tailed Student's *t* test for comparing two groups or one-way ANOVA test for comparing more than two groups.

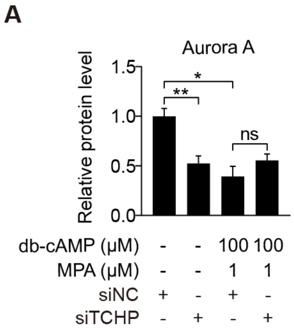

Figure S2. Trichoplein act as the activator of Aurora A**.** **(A)** Western blot quantification of Aurora A after stromal cells were transfected with siTCHP for 24 h then in vitro decidualization for 48 h. In (**A**), data are presented as means ± SD from three independent experiments. ns, (P>0.05), * P<0.05, and ** P<0.01 by two-tailed Student's *t* test for comparing two groups or one-way ANOVA test for comparing more than two groups.

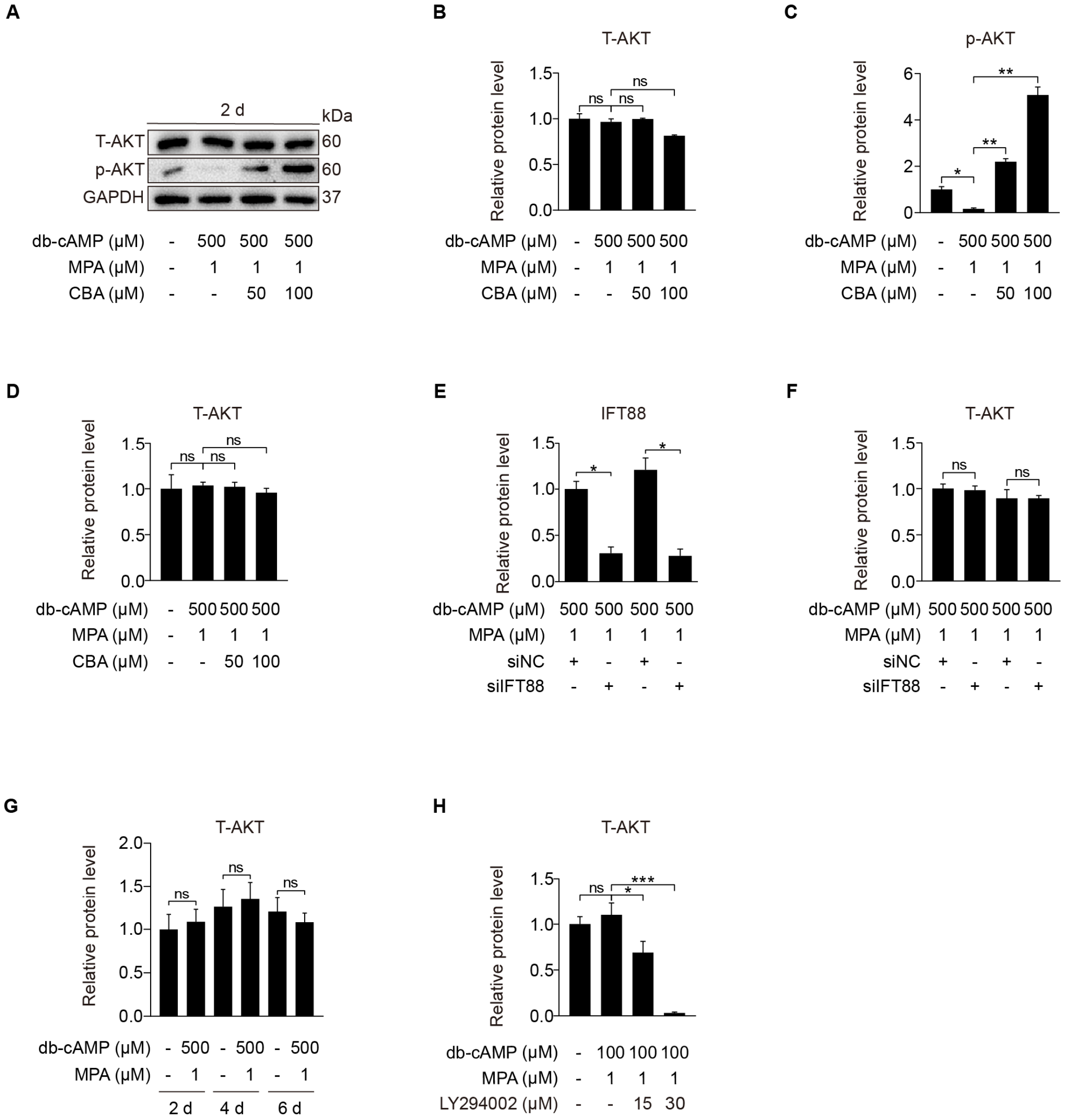

Figure S3. Primary cilia negatively control AKT activation **(A** to **C)** Western blot and quantification of T-AKT and p-AKT (S473) after stromal cells were treated with CBA for 24 h during in vitro decidualization for 2 days. **(D)** Western blot quantification of T-AKT after stromal cells were treated with CBA for 24 h during in vitro decidualization for 4 days. **(E** and **F)** Western blot quantification of IFT88 and T-AKT after stromal cells were treated with siIFT88 for 24 h then in vitro decidualization for 2 and 4 days. **(G)** Western blot quantification of T-AKT in stromal cells during decidualization for 2, 4, and 6 days. **(H)** Western blot quantification of T-AKT in LY294002-treated stromal cells for 48 h. In (**A**), the representative images of three biologically independent experiments are shown. In (**B-H**), data are presented as means ± SD from three independent experiments. ns, (P>0.05), * P<0.05, ** P<0.01, and *** P<0.001 by two-tailed Student's *t* test for comparing two groups or one-way ANOVA test for comparing more than two groups.

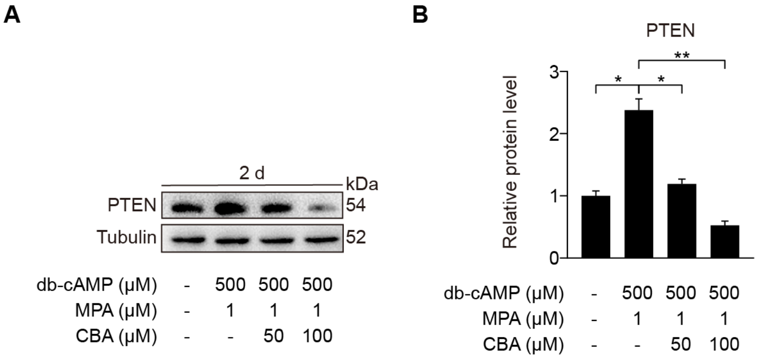

Figure S4. PTEN antagonizes AKT during stromal cells decidualization. **(A** and **B)** Western blot and quantification of PTEN after stromal cells were treated with CBA for 24 h during in vitro decidualization for 4 days. In (**A**), the representative images of three biologically independent experiments are shown. In (**B**), data are presented as means ± SD from three independent experiments. * $P<0.05$ and ** $P<0.01$ by two-tailed Student's *t* test for comparing two groups or one-way ANOVA test for comparing more than two groups.

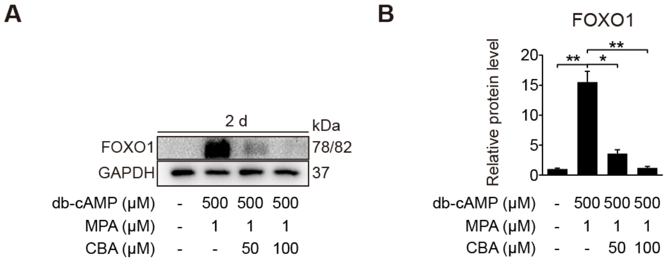

Figure S5. Primary cilia modulate FOXO1 expression by suppressing AKT activity. **(A** and **B)** Western blot and quantification of FOXO1 after stromal cells were treated with CBA for 24 h during in vitro decidualization for 2 days. In (**A**), the representative images of three biologically independent experiments are shown. In (**B**), data are presented as means ± SD from three independent experiments. * $P<0.05$ and ** $P<0.01$ by two-tailed Student's *t* test for comparing two groups or one-way ANOVA test for comparing more than two groups.

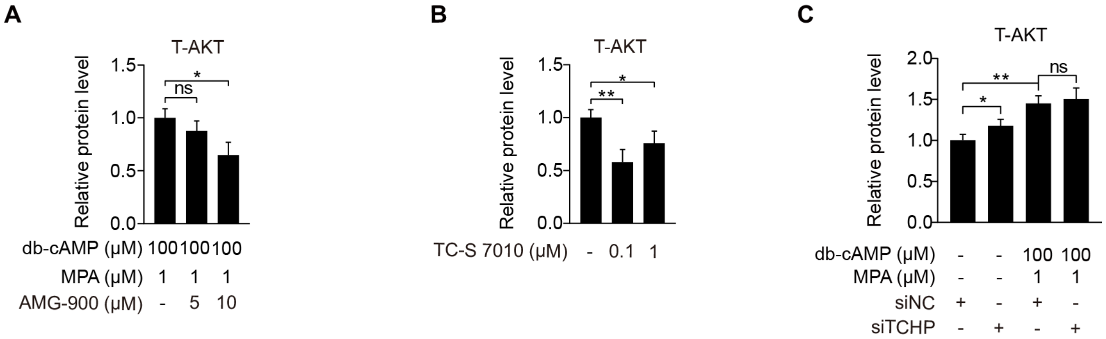

Figure S6. Trichoplein-Aurora A-mediated cilia disassembly contributes to PTEN-AKT-FOXO1 signaling. **(A)** Western blot quantification of T-AKT in AMG-900-treated stromal cells for 48 h. **(B)** Western blot quantification of T-AKT in TC-S 7010-treated stromal cells for 48 h. **(C)** Western blot quantification of T-AKT after stromal cells were transfected with siTCHP for 24 h then in vitro decidualization for 48 h. In (**A**), (**B**), and (**C**), data are presented as means ± SD from three independent experiments. ns, (P>0.05), * P<0.05, and ** P<0.01 by two-tailed Student's *t* test for comparing two groups or one-way ANOVA test for comparing more than two groups.
